# New Attempts to Define and Clarify Lupus

**DOI:** 10.1007/s11926-019-0810-4

**Published:** 2019-02-26

**Authors:** Marta E. Alarcón-Riquelme

**Affiliations:** 1 Medical Genomics, GENYO. Centre for Genomics and Oncological Research: Pfizer/University of Granada/Andalusian Regional Government, Av de la Ilustración 114, PTS, 18016 Granada, Spain; 2 Unit of Inflammatory Chronic Diseases, Institute of Environmental Medicine, Karolinska Institutet, 17777 Solna, Sweden

**Keywords:** Stratification, Systemic lupus erythematosus, Classification criteria, Transcriptome, Epigenetics, Methylation

## Abstract

**Purpose of Review:**

The purpose is to discuss the advances that genetics and genomics have provided to better understand the molecular mechanisms behind SLE and how to solve its heterogeneity. I propose new ideas that can help us stratify lupus in order to find the best therapies for each patient, and the idea of substituting clinical diagnosis with molecular diagnosis according to their molecular patterns, an idea that may not only include lupus but also other diseases.

**Recent Findings:**

The study of rare mutations may provide insight into groups of lupus patients where type I interferon signature is important and help understand those with an atypical clinical presentation. Recent papers used longitudinal blood transcriptome data correlating with disease activity scores to stratify lupus into molecular clusters. The implication of neutrophils in the risk to develop nephritis was established, but also that neutrophils and lymphocytes may correlate with activity differentiating the mechanisms of flares and separating patients into clinically separate groups.

**Summary:**

The role of type I interferon signature is important; however, the stratification of SLE patients according to the genes and cellular compartments being modulated during disease activity may be even more important to define those patients who may benefit the most with new anti-type I IFN receptor therapies.

## Introduction

The heterogeneity of SLE has had for many years an enormous impact in how the disease is diagnosed, but also how patients are to be treated or how research results are reported. SLE is a disease that begins insidiously and some of its manifestations are highly unspecific.

We are still at early stages in understanding how we can classify and stratify SLE patients into groups of molecular clusters, and we are dependent on the clinical parameters at hand: classification criteria or even disease activity indexes or damage indexes, many times non-measurable, subjective, and semi-quantitative parameters that limit our possibilities of using molecular tools more precisely. Here I provide an update on the use of molecular tools to stratify SLE patients into clinically relevant groups. We believe this information will be very important in the use of new biological drugs and will show the molecular patterns of patients who will best benefit from the available treatments, while helping us focus in the discovery of new drugs for the real orphan ones.

Despite the progress in unifying patients with SLE using clinical criteria that have seen several changes in the last 50 years, SLE is still a very heterogeneous disease, a fact that suggests that indeed there may be multiple pathways lying behind disease phenotypes and progression. This is mostly evidenced when it comes to clinical trials, when end points or response needs to be defined. Only some 25% of SLE patients develop glomerulonephritis, and not all patients show an interferon signature. The type I interferon signature is defined by the expression of a large group of genes that characterize downstream events of type I interferon signaling (IFN-I, mainly IFNα and IFNβ), originally described in SLE [1, 2] and later in other diseases [3]. In fact, while pediatric SLE patients show a high prevalence of both (70% of the patients have an interferon signature), adult patients do not, and in many instances, ethnicity and socio-economic aspects are also important in defining the type of disease they present [4, 5]. Therefore it is becoming increasingly important to predict the type of molecular trajectory that an SLE patient will develop. In fact, I may argue that a clinical diagnosis such as SLE limits the possibilities of defining the molecular pattern of an individual, as many such molecular patterns, particularly related to inflammatory processes, may be shared across several diseases (not limited to autoimmune conditions). The definition of normalcy may be changed to whether an individual with a given pattern of gene expression or epigenomic changes may be at risk or not of developing a certain condition. Here I suggest the term molecular diagnosis, a term that will have to be developed with time and as studies advance in defining molecular patterns, particularly in longitudinal studies. Here we still limit ourselves to SLE, and below I will discuss the attempts to molecularly stratify the disease that may help in predicting severe and non-severe trajectories. Nevertheless, I also refer to the recent review where the molecular stratification of several systemic autoimmune diseases is suggested [6].

## Towards a Molecular Stratification of SLE—First Steps: the Role of Rare Mutations

The importance of the stratification of patients into groups that may be amenable to personalized therapies and future studies on the pathophysiology of the disease may find help from the advances in genetics and genomics and eventually should surpass the limitations that some routine laboratory tests and clinical criteria have by deepening the possibilities of precise classification into molecular entities to treat patients based on molecular phenotypes and mechanisms. Such molecular phenotypes are primarily based on blood transcriptome studies, but could very well be complemented with genetic studies. In the case of SLE, many atypical phenotypes that are known may be related to the presence of rare mutations leading to clinical manifestations that combined with other “autoimmunity” tests (e.g. presence of ANA), show features similar to SLE. Indeed, an important set of rare diseases called interferonopathies [7•] have been described and these are individuals with rare mutations showing abnormalities in various sorts of endonucleases and other nucleic acid metabolism enzymes leading to the presence of an interferon signature.

One example is the Aicardi-Goutieres syndrome, a congenital encephalopathy caused by mutations in a gene called *TREX1* [8]. TREX1 encodes for a 3′–5′ exonuclease that prevents the accumulation of nucleic acids inside the cell. Such accumulation leads to the activation of innate immune responses to viruses and the production of type I interferon (IFN) resembling congenital viral encephalitis. Adult SLE patients with heterozygous *TREX1* mutations may have a type of relatively rare skin manifestation called chilblain lupus [9]. It has been recently observed that a larger proportion of neuropsychiatric SLE patients have *TREX1* mutations [10]. Other genes found in rare monogenic interferonopathies are *RNASEH2*, *ADAR*, *IFIH1*, or *SAMHD1*. A somewhat different type of mutation was recently found in the STING gene [11•] in patients with chilblain lupus. A gain-of-function mutation leading to the excessive function of a nucleic acid sensor STING results in constitutive type I IFN activation [11•].

Since many years, it has been known that deficiency of the classical pathway components, through deletion, polymorphisms, or insertions lead to autoimmune-like features. In particular, C2 deficiency leads to SLE-like disease in about 34% of the patients [12]. Most patients with C1q deficiency develop skin and renal manifestations, and around 20% develop neuropsychiatric SLE. C1q deficiency is extremely rare; however, patients with C1q deficiency have elevated levels of type I IFN in the cerebrospinal fluid [13] due to the lack of control of type I IFN production induced by immune complexes.

We recently described the identification of many rare mutations in Icelandic families with multiple cases of SLE [14••]. By performing exome sequencing on the most distantly related affected individuals from two large families and verifying some of the mutations through genotyping or Sanger sequencing, we identified multiple rare and likely pathogenic variants in 19 genes co-segregating with disease through multiple generations. The genes were mostly enriched in the GO categories of immune system development, lymphocyte activation, DNA repair, and VDJ T and B cell receptor gene recombination. We also found further support using a very stringent aggregate association analysis in sporadic cases for the *FAM71E1/EMC10* locus. Another interesting gene for which we did not find sufficient support was *DCLRE1C*. *EMC10* (ER membrane complex subunit 10) codes for a protein involved in endoplasmic reticulum (ER)-associated degradation and lipid transport. This suggests a potential role of ER stress in the disease process. On the other hand, *DCLRE1C* is involved in double-strand break repair, cellular response to DNA damage stimuli, and chromosome organization. Recessive mutations in this gene cause Omenn syndrome, a severe combined immunodeficiency associated with increased cellular radiosensitivity due to a defect in V(D)J recombination that leads to early arrest of both B and T cell maturation [15]. A recent functional study demonstrated that Artemis-deficient cells have type I and type III IFN signatures due to the chronic accumulation of DNA [16].

## The Role of the Transcriptome and the Epigenome in the Molecular Stratification of SLE

Transcriptome and epigenome analyses have been the major source of data with which studies on disease stratification have been based on. Many studies have used blood, and others have used blood-derived cells, primarily T cells, in the case of SLE studies. Others have used tissues, primarily kidney. When using blood, the major problem is the dilution of the signal, if there is a cell-specific transcriptome difference that is searched for. So blood transcriptome analyses provide a general picture. Tissues would be most desired when also blood transcriptome and specific cells are available. There are logistic problems with this, and for SLE, no studies have attempted this, and less so in longitudinal studies. So most studies available have been performed in time windows using either blood, or a type of cell or maybe two, or a tissue.

Bradley et al. using transcriptome data from purified T cell found subgroups of SLE patients according to disease severity [17]. However, the number of patients is very low suggesting overfitting in the data. Flint [18] also investigated the interferon signature in more detail, primarily looking for differences in the type of signature expressed by various cell types (neutrophils, CD4^+^ T cells, CD8^+^ T cells, and monocytes) across four different immune-mediated conditions, including SLE, and a healthy control group. These authors used the weighted gene expression network analysis (WGCNA), which identifies gene modules in the data based on co-expression (a method widely used after the publication of Chaussabel et al. in 2008 [19]). One module was selected for each cellular population as the most representative interferon signature based on gene composition, correlation with SLE diagnosis and correlation with a 21-gene core interferon signature expression profile. An extensive analysis of 1150 genes unique to myeloid subsets and 11 genes unique to T cells was performed to compare between several autoimmune diseases and cellular populations. Examining the median expression of a selected group of type I interferon genes, most of them were found to be highly expressed in myeloid cells and neutrophils, whereas only a few of them had increased expression in T cells. However, higher expression of the T cell-specific modules seemed to be an exclusive feature of SLE, unlike monocytes and neutrophils, which presented similar expression levels across other diseases and controls. As the authors discuss, the similar neutrophil interferon signature across conditions seemed to be concordant with the importance of basal type I interferon signaling in maintaining myeloid populations, whereas it does not seem to be necessary in T cells. On the other hand, the specific expression of T cell modules in patients with SLE are in agreement with findings regarding hypomethylation of type I interferon genes in naive CD4^+^ T cells [20], and the findings on the type II IFN dysregulation mentioned in section I. These results suggest that type I interferon T cell signaling, possibly secondary to IFN-g-induced signaling, might contribute to the development of SLE.

One clear example is the finding that naïve CD4+ T cells from SLE patients are poised to express, that is, prior to stimulation, type I IFN-inducible genes [21].

McKinney [22] separated various cell populations from SLE patients and found that the transcriptome from separated CD8+ T cells would help group SLE patients into two relevant subgroups with different prognoses. The subset of genes defining the poor prognostic group was enriched for genes involved in the interleukin-7 receptor (IL-7R) pathway, T cell receptor (TCR) signaling, and those expressed by memory T cells with a concurrent expansion of CD8+ memory T cells.

DNA methylation is considered the most characteristic epigenetic mark [23]. Its presence or absence in different regulatory regions of the genome has been associated with changes in gene transcription, chromosome stability, or the regulation of alternative splicing [24]. Aberrant patterns of DNA methylation have been implicated in autoimmune disorders [25].

Comparing between different autoimmune diseases for discriminant profiles that could be useful in routine clinical practice has been performed. One study showed a clear relationship between SLE and rheumatoid arthritis (RA), which could be classified into three groups with specific profiles that overlapped across diagnoses [26]. Interestingly, they replicated the association between RA and SLE in later studies [27], but not the groups, maybe because of the sample size. They showed that early RA patients grouped better with SLE than RA patients with longer disease duration [28]. The shared gene signature affected B cell function [29], suggesting that these individuals could eventually be treated with therapy targeted to B cells, but also that early in disease, before progressing towards tissue or organ damage, the similitudes are larger.

Another study analyzing jointly SLE and systemic sclerosis (SSc) transcriptomes found that 62% of differentially expressed genes in SSc versus healthy individuals were also differentially expressed when analyzing SLE [30]. Type I IFN-inducible and JAK/STAT signaling pathways were enriched, as well as pathogen pattern molecular recognition functions. They also found that some SSc patients grouped with the SLE patients. These “lupus-like” patients had increased type I IFN-inducible and plasma cell gene expression. Increased expression of IFN-inducible genes was related to disease activity for both diseases, and a positive correlation with presence of antinuclear antibodies [31], already observed by others [30]. The type I IFN signature was also found associated with subsets of RA [32]. A study searching for shared signatures between autoimmune diseases [33, 34], showed that genes differentially expressed between systemic autoimmune diseases and controls are common across diseases, primarily, but not exclusively, the interferon signaling pathway. From the clinical point of view, this information could be important in the potential use of anti-IFN receptor therapy (Anifrolumab) not only for SLE and Sjogren’s syndrome (SjS), but potentially also for subsets of RA and SSc once the molecular patterns characterizing the different patients are available [6].

Early studies on DNA methylation comparing RA and SLE showed similar patterns of global hypomethylation in T lymphocytes, synovial tissue, synovial mononuclear cells, and peripheral blood [35, 36]. In genome-wide studies, this global T lymphocyte hypomethylation was confirmed for SSc and SjS [21, 37]. Interestingly, this pattern was not observed in other inflammatory diseases, such as dermatomyositis [37]. In SLE, SSc, and SjS, the hypomethylated pattern was observed in B lymphocytes, monocytes, dermal fibroblasts, and leukocytes [38–40]. These were correlated with a decrease in expression of DNA methylation machinery genes *DNMT1*, *DNMT3B*, or *MBD4*. In SLE and SjS, hypomethylated genes are enriched with the type I interferon pathway genes [21, 38, 41]. It is notable to mention that methylation machinery genes are dependent on environmental factors such as dietary folates [42, 43]. On the other hand, the variety of cells showing the type I IFN signature suggests that whichever inducer there is, it is systemic, possibly viral. A recent study showed that 50% of the promoters of known genetic risk loci of SLE are occupied by the Epstein-Barr virus EBNA2 protein, many of which cocluster with other human transcription factors. This is a first example of a gene-environment interaction with direct potential epigenetic regulation and conditioning of the effects of the genetic risk loci by a viral transcription factor [44••].

*IFI44L* is part of the type I IFN signaling response found in SADs. The *IFI44L* promoter was hypomethylated in SLE patients. When comparing with RA and SjS, despite the IFI44L promoter being hypomethylated in all diseases, the methylation levels distinguished SLE from the other [45]. Another study compared DNA methylation in monozygotic twins discordant for three diseases (including SLE and RA). The authors only found methylation differences in SLE twins, with 49 differentially hypomethylated genes in cases [40].

One issue that is of major interest is what is the gene expression response during disease activity and if this may stratify patients or reveal different patient groups or classes.

On this regard, Banchereau, et al. [46••] used, for the first time, longitudinal total blood gene expression data and identified seven groups of SLE patients where specific gene expression modules were associated with each cluster [46••].

The weighted gene expression network analysis (WGCNA) that identifies gene modules in the data based on co-expression was the method used [19]. Interestingly, they selected the most correlated module for each patient with SLEDAI and projected their expression profiles to the Chaussabel modules [19]. This is problematic to understand because many of the non-selected modules were also highly correlated, and therefore, many genes potentially useful in the stratification procedure could have been missed. The seven groups of patients described corresponded to five immune signatures that were different from each other in terms of the types of cellular mechanisms: lymphoid, erythropoiesis, plasma cell, neutrophil/myeloid, and type I IFN. However, at least three of the groups of patients had an IFN module and it is difficult to understand why only one module was selected as many others were still highly correlated.

No differences were observed across the groups in terms of demographic parameters, with the exception of one group where all patients had nephritis and correlated with the IFN, neutrophil, and plasmablast-associated modules. These patients had the most severe disease and the presence of anti-dsDNA antibodies. In addition, the neutrophil and IFN signatures correlated strongly with development of lupus nephritis and were modified by Mycophenolate Mofetil (MMF) treatment, particularly in patients with proliferative rather than membranous glomerulonephritis.

A latest attempt was performed by us, using the data from Banchereau, et al., and an extra set of adult SLE patients from Johns Hopkins [47••]. Instead of taking a module to define the transcriptome stratification, individual genes were selected by their correlation with the SLEDAI, followed by clustering. The WGCNA was then used to investigate the functionality of the genes within each cluster. This study gave three clusters that were replicated in the adult set. It should be noted however that the SLEDAI is a semi-quantitative score with many drawbacks and that a continuous score based on real measurements that may be correlated with transcriptome data in a longitudinal fashion would be the ideal. The three clusters had particular characteristics: cluster 1 was heterogeneous and contained features of clusters 2 and 3, but the patients from that cluster could not be assigned to any of the other two. Cluster 2 was very differentiated and showed a clear relationship with the type I IFN signature. This signifies that during disease activity, with higher SLEDAI scores, the type I IFN signature genes correlated positively with the score. There was also a correlation with increases in the levels of neutrophils, C3, and the ESR and a negative correlation with the levels of lymphocytes. In adult data, this cluster was also associated with lymphopenia. On the other hand, cluster 3 was completely opposite, showing instead a positive correlation between SLEDAI and lymphocytes and a negative correlation between the SLEDAI and neutrophil numbers or proportions. Interestingly, in pediatric patients, the definition of the three clusters was very clear, less so in the adults.

However, this has two clear explanations that adults SLEDAI scores were much weaker and showed less variation than in the pediatric patients, and less adult patients were available. To perform the analyses, two conditions need to be met: a variable SLEDAI and at least three time points of transcriptome data to perform the correlation, which makes this sort of study somewhat difficult. In addition, a somewhat large number of patients (the study used 80 pediatric of the Banchereau study and 65 adults) is needed to meet the statistical requirements.

Clinically, very interesting differences were observed. Patients from cluster 1 had the highest risk to develop proliferative nephritis while those from cluster 3 had a very low risk. This was particularly obvious in adults where 65% of the patients from cluster 1 were at risk to develop proliferative nephritis against 13% of cluster 3. Cluster 2 also had a high risk to develop proliferative nephritis, but somewhat lower. Patients from cluster 3 showed features of skin disease, antiphospholipid syndrome, and enhanced liver enzymes. Disease activity did not condition the clusters, and indeed, there were no differences in the SLEDAI components or in the magnitude of the components between clusters. Also, when treatment was verified, it was observed that this did not condition the assignment of the patients into the clusters, and again, no differences in treatment between the groups were observed. Furthermore, as neutrophils appeared to be important in defining the clusters, there were no differences in neutrophil numbers between the groups after treatment. In fact, when performing a feature (gene) selection against treatment doses, for each type of therapy for which the data was available, only 2% of the genes selected overlapped with the genes selected when correlating with the SLEDAI. In summary, the clusters had molecular patterns that most likely reflected the drivers of the disease activity, being primarily differentiated by the cell types: neutrophils on the one hand, and lymphocytes on the other.

It is certainly possible that once the groups have been stratified into molecular blood patterns, patterns in tissues may create a sub-stratification of each cluster, and such sub-stratification may be important in the treatment of, for example, lupus nephritis. I believe this would be the case in SLE, particularly with the differences we observe in the risk to develop nephritis in clusters 1 and 2. However, cluster 3, a cluster formed by individuals with secondary Sjogren’s syndrome and anti-phospholipid syndrome, may still develop other classes of glomerulonephritis.

One important point here is that patients having an IFN signature in a one-time window do not necessarily respond with changes in expression of interferon genes during moments of disease activity or inactivity. Thus, simply taking patients with high or low IFN gene signature expression at one time point may not be reflecting the reality of the process occurring during flares.

## Conclusions

Many questions are open still and would require the possibility to study SLE patients longitudinally and obtain peripheral blood cells and cell patterns, something possible today using mass cytometry methods and single cell methodology. Another important aspect is the possibility to obtain tissues to investigate the development of nephritis in parallel to the longitudinal data collection to be able to identify markers that may predict the development of nephritis. A disease classification using one time point may be difficult, as a follow-up of disease progression seems to be necessary to stratify the patients, but may reduce the time a patient may undergo unspecific therapy such as corticosteroids, or depending on the severity at presentation, immunosuppressive therapy, and move the patient rapidly to a more personalized treatment once the acute need is covered.

Among the future analyses to be performed in the near future would be to observe if using the patient stratification, different drugs are detected that follow a given pathway in relation to the expressed genes. For this, drug repurposing or repositioning analyses can be done using the LINSCLOUD platform [48] in its newest version, CLUE.

Finally, one important aspect is the idea that molecular patterns may be shared across several diseases, as described above. Thus, instead of a clinical diagnosis, we would ascribe a molecular diagnosis and a personalized treatment in accordance.
